# Robust automated method of spatial resolution measurement in radiotherapy CT simulation images

**DOI:** 10.1002/acm2.70006

**Published:** 2025-02-13

**Authors:** Pavel Govyadinov, Rick. R. Layman, Tucker Netherton, Raymond Mumme, Aaron. K. Jones, Laurence. E. Court, Moiz Ahmad

**Affiliations:** ^1^ Department of Radiation Physics The University of Texas MD Anderson Cancer Center Houston Texas USA; ^2^ Department of Imaging Physics The University of Texas MD Anderson Cancer Center Houston Texas USA; ^3^ The University of Texas MD Anderson Cancer Center UTHealth Houston Graduate School of Biomedical Sciences Houston Texas USA

**Keywords:** CT, image reconstruction, image quality assessment, spatial resolution, quality control

## Abstract

**Background:**

Variation in imaging protocol, patient positioning, and the presence of artifacts can vary image quality in CT images used for radiotherapy planning. Automated methods for spatial resolution (SR) estimation exist but require further investigation and validation for wider adoption.

**Purpose:**

To validated previously existing algorithm for SR estimation and introduce improvements that make it robust to patient positioning, CT protocol, site, and artifacts.

**Method:**

A reference algorithm based on the previous gold standard was recreated and modified to improve robustness. The algorithms were tested on three different datasets: (1) a cylindrical ACR CT QC phantom scanned using a Siemens SOMATOM Definition Edge scanner and reconstructed using 61 different kernels, (2) a set of anthropomorphic phantoms scanned with the presence of artifacts common to clinical acquisitions such as blankets and immobilization devices, and (3) a clinical patient dataset of head and neck (HN) CT scans (nine patients) and spine/pelvis (10 patients). The robustness of both algorithms was tested on the clinical patient data.

**Results:**

Over the range of tested kernels, both algorithms were accurate when the ground truth MTF f_50_ was within the range 0.2–0.7 mm^−1^ in the cylindrical phantom datasets with an RMS error of 10.3% and 3.8% for the reference and modified versions of the algorithm, respectively, as compared to the ground truth. In the anthropomorphic phantom datasets the reference algorithm showed an 8.4% and 30.0% difference from ground truth for the Pelvic and HN phantoms, respectively, while the modified algorithm showed 4.9% and 3.9% percent difference from ground truth. In the clinical dataset the reference algorithm estimated a mean f_50_ value of 0.21 ± 0.03 mm^−1^ and 0.25 ± 0.03 mm^−1^ for pelvis/spine while the reference algorithm estimated mean of 0.28 ± 0.02 and 0.29 ± 0.01 mm^−1^ for HN and pelvis/spine, respectively, as compared to the ground truth found to be 0.28 mm^−1^ on the cylindrical phantom.

**Conclusion:**

The SR algorithm was validated cylindrical/anthropomorphic phantoms and clinical CT scans. Further modifications were tested and showed improved accuracy in more challenging CT acquisitions.

## 1 INTRODUCTION

Computed tomography (CT) image quality characteristics, such as spatial resolution (SR), noise, and contrast, are generally characterized using phantoms as part of quality control (QC). Clinical image quality is inferred from the QC results. However, both the imaged subject and imaging parameters in phantom‐based image quality tests may differ from clinical imaging in ways that can affect clinical applications, such as auto‐contouring in radiation therapy.^1^, ^2^ Variability in imaging protocols further confound the issue resulting in statistically significant variations in image quality among imaging facilities.^3^ Moreover, many clinics may not have the phantoms, personnel, and resources to perform QC measurements of certain image quality metrics, exacerbated by a lack of a unified image acquisition standard.^4^, ^5^, ^6^ There is considerable interest in remote automated treatment planning especially to assist radiation oncology in low and middle‐income countries (LMICs). In such setting, quality control on acquired CT simulation images at a remote LMIC site is a significant and challenging problem. For instance, automated treatment planning may use automated anatomical segmentation, and the quality of the segmentation may depend on the quality of CT simulation images.^1^, ^7^ Therefore, there is a need to develop automated QC and standards to assure consistent and high‐quality clinical images required for clinical applications such as auto‐contouring in radiation therapy. Currently there are a multitude of automated methods available for calculating SR with high precision using cylindrical phantoms^8^, ^9^, ^10^ and a typical QC process for CT scanners involves regularly performing phantom tests to ascertain if the device is performing within a tolerance range. Such QC, however, assumes ideal imaging conditions that may not account for subject‐specific variations. Nor is it practical to test a multitude of clinically used imaging protocols with phantom tests.

A method of measuring the SR metric of modulation transfer function (MTF) directly in clinical CT images has been previously presented^11^ and extended to radiologist preference^12^ and mathematical observer.^13^ The method measures the sharpness of the skin‐air interface within CT images. Although successfully applied to diagnostic CT examinations, the unique challenges associated with radiotherapy CT simulation has not been reported. Application to CT simulation of radiotherapy poses unique challenges. Specifically, the presence of immobilization devices common to radiotherapy may interfere with the assessment of the skin‐air interface and produce erroneous measurements.

The purpose of this study was to improve and validate automated techniques for evaluating the SR of clinical CT imaging, paying special attention to robustness over patient setup commonly used in radiotherapy CT simulations. For instance, clinical data are subject to a multitude of artifacts not present in ideal, uniform phantoms, such as from clothing, immobilization devices, and pacemakers. An update of a previously published reference method of Sanders et al. was implemented and tested in various phantoms and clinical radiotherapy CT simulation image sets. This work provides several important algorithmic contributions that robustly filter out invalid samples of the patient skin to air edge‐spread function. These contributions are shown to improve resolution measurement accuracy over the reference method in challenging clinical conditions, including attenuating external markers and immobilization devices. In addition, the robustness of the method across widely varying image reconstruction filters was demonstrated on a phantom.

## 2 MATERIALS AND METHODS

A brief review of the reference method of Sanders et al. is provided. Given a set of CT images, the reference method first segments the patient from the external space (air, CT table) using Otsu thresholding.^14^ The volume is meshed using a tetrahedral volumetric mesh and the air–skin interface is designated as the tetrahedrons on the surface of the mesh. The edge spread function (ESF) of the skin‐air interface is sampled at a multitude of points along the surface mesh. A tail‐replacement procedure is used to correct varying skin image intensity with depth; the resulting ESFs are normalized, centered, and aggregated into a global ESF. A regularization procedure smooths the ESF and eliminates spurious signals. Finally, a Fourier transform of the regularized global ESF produces a global modulation transfer function (MTF) characterizing the CT scan resolution.

The reference method was implemented with key modifications. The meshing strategy was updated to use marching cubes^15^ to generate a triangular mesh and extract the air‐to‐skin interface. We implemented additional computationally low‐cost filters to remove invalid individual ESF curves. We detected extraneous objects such as immobilization masks in the ESF tail. Additionally, during the logistic function fit we implemented simple thresholding‐based detection to remove the incorrect ESF curves. Additionally, we trim the tails directly in the globally aggregate oversampled ESF curves since the tails hold no information about the transition through air‐to‐skin.

### 2.1 Algorithm description

Given a set of CT images, the algorithm extracts the air–skin interface mesh using the marching cubes on an Otsu mask generated from the CT dataset. Due to the nature of marching cubes, each element of the resulting mesh is smaller or equal to the size of the voxel. This mesh is then over‐sampled in head and neck (HN) patients to increase the number of ESF samples. Perpendicular Bresenham lines^16^ are generated from the center of each triangle mesh component. Image pixel values are sampled along the Bresenham lines without the use of interpolation to generate a set of individual edge spread function (ESF) curves. The distance of each pixel in the ESF from the skin‐air interface was calculated using a kdtree, identical to Sanders et al.

**FIGURE 1:**
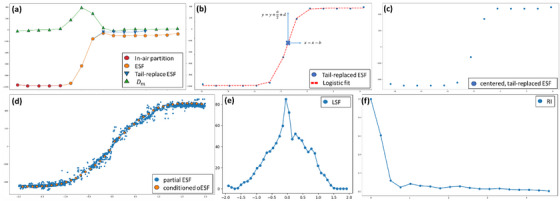
Visual description of the algorithm. Each individual ESF curve is tail‐replaced (a), centered (b, c). The curves that pass the filters are then binned and conditioned to generate the oESF curve (d), a derivative LSF (e) and an fft is applied in order to generate the RI (f). The ESFs and all further derivatives are for extracted from a spine patient in the clinical data presented in this paper.

Each ESF curve undergoes a tail replacement and centering operations, and a set of filters is applied to remove invalid ESF curves. The remaining ESF curves are aggregated using binning and regularized to generate a single oversampled ESF (oESF). From the oESF, the algorithm computes a line spread function and modulation transfer function (MTF). Finally, we return the calculated f50 value of the MTF. The pseudo‐code for the algorithm is provided in Algorithm 1 and a visual example is presented in Figure 1:

ALGORITHM 1 **Modified spatial resolution approximation**

**Table:** 

**Input**: A stack of CT images in DICOM format **C** with voxel spacing s_x_, s_y,_ s_z_:	
M = GenerateOtsuMask(**C**)	(generated a masked volume)
Surface = MarchingCubes(M)	(generate a surface mesh)
**for** every triangle *t* in Surface **do**	
l **=** BresenhamLine (normal to t)	(generate Bresenham line)
ESF = SampleVolume(C, l)	(sample **I** intersect **C**)
ESF’ = precentering_filters(ESF)	(apply pre‐centering filters)
ESF_centered_ = center(ESF^'^)	
ESF’_centered_ = postcentering_filters(ESF_centered_)	(apply post‐centering filters)
**end**	
oESF = binning(0.1*s_x_)	(generate oESF)
oESF’ = isotonicRegression(oESF)	(apply smoothing)
LSF = ∆oESF’	(calculate LSF)
MTF = fft(LSF)	(calculate MTF)
**Return** calculated MTF(f50)	(calculated f50)

### 2.2 Mesh generation

Mesh generation is done using pyvista, a pythonic Visualization Toolkit implementation.^17^ The meshes are smoothed to deal with larger voxel sizes. Smoothing is performed using the Taubin algorithm to remove high curvature variations while preserving the total volume.^18^ We perform 100 iterations of the algorithm with a band pass filter coefficient of 0.05.

In all cases, the dataset is truncated. We crop 10 mm at both longitudinal ends of the image volume to avoid calculation errors from insufficient out of axial plane sampling. Additionally, we crop the dataset to ensure that the individual curves do not intersect with the table. This is done by calculating the center point of the reconstructed volume and projecting a horizontal plane approximately 50 mm away from the center point toward the table. The volume is cropped below that plane. The direction of all the crop operations and the normal of the plane is defined by the image coordinate system defined in the DICOM image metadata. (We noted the crop plane distance as approximately 50, since the actual distance away from the centerline was 50 pixels multiplied by the x, y pixel spacing parameter, which was 500‐mm/512 pixel = 0.977 mm/pixel in nearly all our clinical scans).

### 2.3 Robust filtering

In addition to the filters presented in Sanders et al., our method uses additional filters to remove invalid individual ESF curves prior to aggregation. These filtering routines are broken up into two stages: pre‐centering and post‐centering.

An individual ESF curve $E_{m}\left(x\right)$ is composed of a set of *N* samples $X\;=\{x_{0}\text{…}x_{N}\}$. During the tail‐replacement stage of the algorithm, the set X is decomposed into three sets: $X_{air}=\left\{x_{0.}\text{…}x_{a_{0}}\right\},\;X_{transition}=\left\{x_{a_{s}}\text{…}x_{a_{e}}\right\},\;X_{tissue}=\left\{x_{a_{e}}\text{…}x_{N}\right\}$. Where $x_{0},\;x_{a_{s}},x_{a_{e}},x_{N}$, are the first, first transitional, last transitional and last samples in the ESF curve, respectively. $X_{air},\;X_{transition},X_{tissue}$ are identified using a transformation of $\Delta E_{m}$ to a function $D_{m}$ (Equation 1).

(1) $$\begin{array}{cc} D_{m}\;\left(x\right) & =\sqrt{\Delta E_{m}{\left(x\right)}^{2}+{\bar{\Delta E_{m}}}^{2}}-\bar{\Delta E_{m}}\\ X_{transition} & =\{x_{i}|\;D_{m}\left(x_{i}\right)\rangle 20HU\} \end{array}$$

where ∆E_m_ (x) is the first derivative of the m‐th ESF curve, and E_m_ (x) is an ESF curve in Hounsfield Units (HU) as a function of distance. The transformation of ∆E_m_ to D_m_ makes ∆E_m_ sparse by suppressing small values; this helps with robustness of identifying the transition region. The threshold value of 20 HU was found experimentally and is valid under the assumption that the first derivatives in the air and tissue tails are generally stable.

While this assumption holds for the majority of the ESF curves, an individual ESF curve may include transition between multiple materials, such as mask, blankets, clothing, or ancillary regions. For such cases, we implement additional filters that check air, tissue, and transition regions for incongruencies. We also account for errors in surface–air interface segmentation. All pre‐centering filters and their use cases are identified in Table 1. All other curves proceed to the tail‐replacement stage of the algorithm.

**TABLE 1:** Table describing the filtering performed on each individual ESF curve.

Pre‐centering		
In air artifact media filtering	$\sum_{x_{0}}^{x_{a_{s}.}}\Delta E_{m}\left(x\right)<-100$	Detects extraneous objects in the air tail of each individual curve
Ancillary region filtering	$\sum_{x_{a_{e}}}^{x_{N}}\Delta E_{m}\left(x\right)<-300$	Detects air‐tissue‐air and similar transitions as well as noisy ESF curves in sharper kernel reconstructions
Transition region check	$\left.\begin{array}{c} \begin{array}{c} x_{n}>N*0.2\\ x_{n}<N*0.8 \end{array}\\ E_{m}\left(x_{n}\right)<E_{m}\left(x_{n+1}\right) \end{array}\right\}\;where\;x_{n}\in X_{transition}$	Checks whether the transition region is roughly in the center of the entire ESF based on the number of samples *N* and the transition region is strictly increasing.
Clothing*	$E_{m}\left(x_{air}\right)<-950$	Detects clothing (in close contact with skin??)
In‐plane normal*	$\cos^{-1}\left(\frac{\vec{x}}{\left|x\right|}\times \vec{\mathrm{xy}}\right)<10^{\circ }$	Detects whether the Bresenham sample passes between slices

*Note*: Filters denoted with an * are present in the foundational work of Sanders et al.

The centering stage of the algorithm is implemented using the scipy *optimize* ^19^ package and verifies correct centering and tail replacements. To bring all ESF curves into the same coordinate space, each set of samples is fitted to a logistic regression function (Equation 2)

(2) $$f\;\left(x\right)=\frac{a}{1+e^{\left(-c(x-b\right))}}+d$$

where *a,b,c,d* are the fitted coefficients initialized to [1000, 0, 1.0, −1000], respectively. The approximated coefficients, *a,b*, and *d* are then applied to center the curve about the origin (Equation 3).

(3) $$\begin{array}{cc} X_{m}^{\prime } & =X_{m}-b\\ E_{m}^{\prime } & =E_{m}-\left(\frac{a}{2}+d\right) \end{array}$$

where $X_{m}^{\prime },$ $E_{m}^{\prime }$ are the location and amplitude values of the transformed m‐th ESF curve. We then test that the curves all exist within a common bounding box, and are not deformed. Full descriptions of the post‐centering filters are defined in Table 2.

**TABLE 2:** A table describing the each ESF inclusion filter performed on each trimmed and centered ESF curve.

Post‐centering		
Bounding box	$\begin{array}{c} {X_{m}}^{\prime }\in \left[-20,20\right]\\ E_{m}^{\prime }\in \left[-600,600\right] \end{array}$	Checks whether the a,b,c,d coefficients have been calculated correctly
Max‐Min curve definition	$\max\left(E_{m}^{\prime }\right)-\min\left(E_{m}^{\prime }\right)\in$ [900, 1200]	Checks whether the air‐to‐skin intensity difference is within the expected range.

### 2.4 Aggregation and post‐aggregation

Once the centering subroutine completes, the algorithm generates the oversampled ESF (oESF) curve from an aggregation of individual ESF curves. The points from every individual centered ESF are binned together, with an aggregate bin value equal to the mean of ESF samples in the bin. The bin size is set to 10% of the pixel size in the *x, y* direction. Additionally, we isolate the center 70% of the curve containing the transition region and eliminate the air and tissue ends prior to the LSF transform. The resulting oESF is then conditioned using isotonic regression^20^ and transformed into a Line Spread Function by taking a derivative. Finally, a Fast Fourier transform is performed in order to generate the Modulation Transfer Function and attain the estimate of the f_50_ SR metric.

### 2.5 Special algorithm for head and neck CT

**FIGURE 2:**
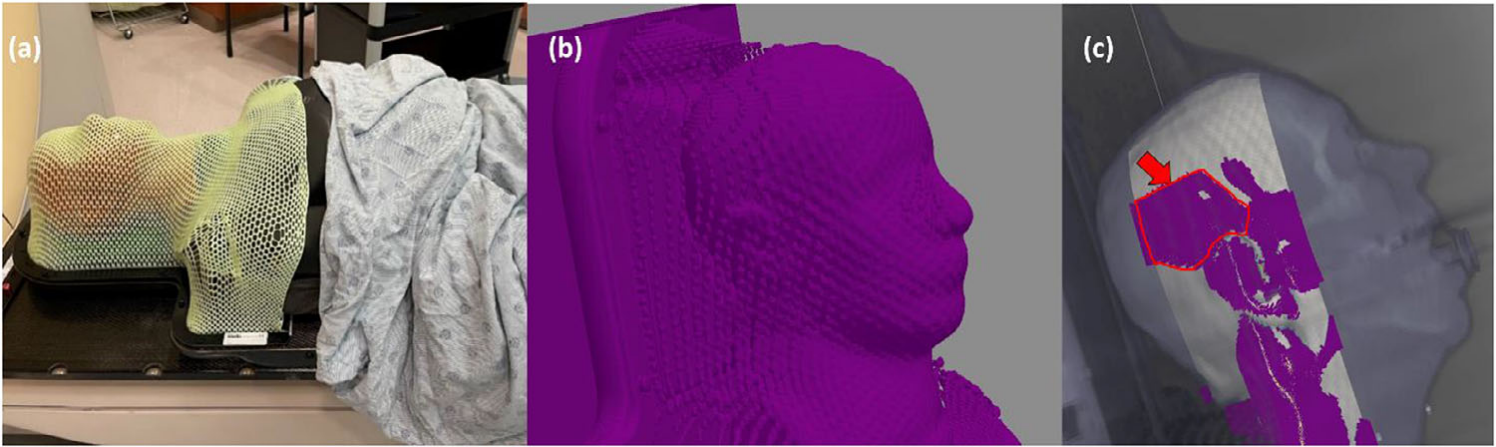
Figure showing the immobilization device problem and our implemented solution. When an immobilization device is present (a), many samples pass through the mask elements which is a part of segmentation (b). To improve the accuracy, we limit the sampling area in the HN protocol to the lateral regions of the head and around the ear (c), where the mask makes less contact with the skin and the mask cells are larger. The arrow in (c) designates samples in the ear region that have passed the in‐plane filter and will be processed by the pre‐centering and post‐centering filters.

Additional modifications were implemented for HN images where the patient is immobilized using a thermoplastic mask. The sampling area is limited by removing any mesh elements posterior and anterior of the ear region (determined to be roughly in the center of the CT image stack). Specifically, we truncate the image volume to a 75 voxel wide slab in the anterior‐posterior direction (Figure 2c). Finally, we oversample the clipped region by a factor of 2 to extract the maximum number of ESF curves from the limited region. This extra operation is done only in the case of the HN image acquisitions.

### 2.6 Uniform phantom

A uniform cylindrical phantom, ACR CT QC phantom: Module 3 – uniformity, was scanned on the Siemens SOMATOM Definition Edge (Siemens Healthineers, Forchheim, Germany) scanner with the following acquisition parameters: 120 kV, 240 mA, 38.4 mm, 500 ms for the Abdomen(protocol) and 120 kV, 400 mA, 38.4 mm, 500 ms for the AbdSeq protocols for values of kV, mA, beam width and rotation time, respectively. Reconstructed images of the phantom were generated with filtered back‐projection algorithms, using 61 different convolution filters and creating 122 data points. These kernels represent the entire range of filters on the system ranging from the smoothest [Br32] to the sharpest [Bl57]. For a full table of filters please see Appendix A

The SR of the resulting reconstructions was evaluated specifically for ACR phantom analysis using: (1) our experimental method and (2) the reference method of Friedman et al. Both methods produce an MTF, and the MTF produced by the Friedman method was taken as ground truth. The MTF‐50 value, the spatial frequency at which the MTF equals 0.5, was derived from both methods The accuracy of the MTF‐50 value was derived from our experimental method and compared to the ground truth.

### 2.7 Anthropomorphic phantom

The experimental method was tested on CT scans of two different customized anthropomorphic phantoms with various devices typically used in radiotherapy CT simulation. These two anthropomorphic phantoms represented two different body regions in an average size adult male: HN, and abdomen & pelvis. The phantoms were manufactured by The Phantom Laboratory (Salem, NY, USA) and scanned using a Brilliance Big Bore Scanner (Phillips CT, Amsterdam, Netherlands). Since our method focuses on the interface between the phantom surface and air, our methods do not depend on the internal contents of the phantoms.

Additional devices were placed external to the phantom to represent typical patient setup in CT simulation. These setups represent challenges to our method which attempts to measure the air–skin interface. For the HN phantom, a custom thermoplastic immobilization mask was placed on the phantom that conformed to the head and shoulder (Figure 2). For the abdomen and pelvis phantom, localization fiducial markers were placed anteriorly and laterally centered on the phantom with a cotton towel folded over onto itself (i.e., two layers) over the groin (Figure 2a). All anthropomorphic phantoms were set up on a flat carbon‐fiber tabletop used in radiotherapy CT simulations.

For both of these scenarios, corresponding scans of a CTDI phantom, a 32‐cm diameter cylinder of acrylic, were acquired using identical imaging parameters (120 kV, 383 mA, 12 mm, 500 ms, UB kernel for kV, mA, beam width, rotation time reconstruction kernel, respectively) as in the anthropomorphic phantom scans. The MTF‐50 value of the CTDI phantom image was calculated using the reference Friedman method and taken as ground truth.

The SR in each of the anthropomorphic phantom scans was calculated using both the Sanders method and our updated method. The accuracy of both of these methods was calculated and compared to the ground truth.

### 2.8 Clinical data

Clinical images of patients undergoing standard of care radiotherapy CT simulation were acquired and used to test our method. Images from nine randomly selected patients receiving CT simulation prior to a course of radiation therapy for HN radiotherapy were accessed. Images from eight randomly selected patients receiving CT simulation for either pelvic radiotherapy and two patients receiving lumbar spine radiotherapy were accessed. The pelvic and spine cases used equivalent setup, immobilization, imaged anatomy, and imaging protocol; therefore, we have grouped these cases together into a cohort of 10 pelvis/spine cases). The image data from these CT simulation procedures were used retrospectively for this research study. The retrospective data collection was approved by our Institutional Review Board and patient informed consent was waived. Patient datasets had diverse demographic, positioning and height and included juvenile patients to test the robustness of the algorithm. All HN patients had a thermoplastic immobilization mask of some kind during acquisition.

Both the experimental and reference methods were used to measure SR (MTF‐50) in these data sets. The MTF‐50 acquired using the Friedman algorithm in the 32‐cm phantom during the corresponding protocol was taken as the ground truth.

### 2.9 Filter sensitivity study

In order to ascertain the sensitivity of our modified algorithm to parameter changes, we performed a study of the sensitivity of our measurement in clinical cases to the various ESF filters used in our method. We examined how many ESF curves each filter removes, and the effect of these filters on the final f50 measurement. Since the filters mimic the swiss cheese model where each consecutive filter is likely to catch any ESF curves the previous filters missed, we adopted an on‐off, one‐all strategy. To do this, we performed the f50 estimation with only a single filter at a time, leaving all the curves that other filters would otherwise catch and eliminate. The rest of the execution proceeded as described in Algorithm 1.

We also studied the effect of resampling the mesh on the resulting f50 approximation. Resampling the mesh has an obvious performance advantage at the cost of accuracy, and the mesh resampling coefficient is the singular tunable parameter during the execution. The adaptive resampling algorithm attempts to retain the total volume of the mesh, by reduce the number of triangle elements by a set percentage. We ran our patient datasets through the entire algorithm using resampling values of 100%–10% in increments of 10%.

## 3 RESULTS

### 3.1 Uniform and anthropomorphic phantoms

**FIGURE 3:**
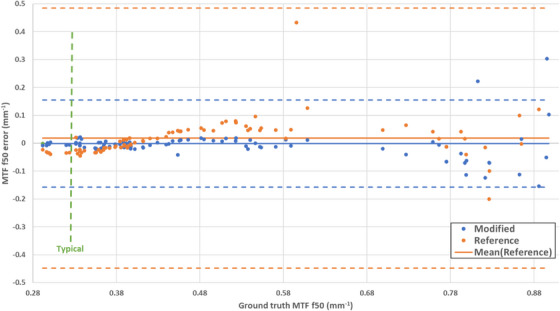
Accuracy of the modified and reference resolution measurement algorithms over a range of image reconstruction kernels with varying spatial resolution (modulation transfer function f50 value). Mean error (solid) and ±2STD (dashed) lines are also plotted. The typical f50 for simulation performed with field of view of 50 cm is labeled on the plot in green.

Both algorithms were accurate when ground truth MTF f50 was within the range of 0.2–0.7 mm^−1^. Twenty out of 122 reconstruction filters were deemed outliers due to their specific use. For example, some of the sharper reconstruction filters designed for highlighting potential anomalies in bone tissue tended to amplify the noise in the phantom‐air (skin–air) interface resulting in incorrect segmentation and noisy ESF curves that were mostly rejected during filtering. With the outliers removed the modified and reference algorithms had a root‐mean‐square error (RMS) of 3.8% and 10.3% with a mean error of 1.5% and 3.5%, respectively. Figure 3 shows the performance of the two algorithms for the reconstruction kernels without the outliers, note that both algorithms became somewhat less reliable when the ground truth MTF f50 was above 0.7 mm^−1^.

The reference algorithm performed well on pelvis/spine CT scans, showing 8.4% difference as compared to the ground truth calculated on the 32‐cm CDTI phantom. On the HN phantom, the reference algorithm had a 30.0% difference, respectively. The modified algorithm performed better, with a 4.9%, 3.9% difference in pelvis and HN, respectively.

### 3.2 Clinical data

**FIGURE 4:**
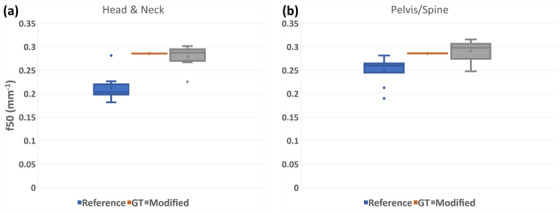
Box plots showing the results of the patient data ran on nine HN scan patients (a) and 10 pelvis/spine scan patients (b). With all the parameters matching between each patient in each group and the respective anthropomorphic phantom, the ground truth (GT) was taken to be at the value computed using the Friedman phantom; represented in orange in each plot.

Box plots in Figure 4 present how the two algorithms performed on the patient datasets in both HN and pelvis/spine groups. With the ground truth set at the Friedman‐calculated value of the cylindrical phantoms of 0.28 mm^−1^ and 0.28 mm^−1^ for the HN and pelvis/spine, respectively, the reference version underestimated MTF in the patient population, with a mean (± population standard deviation) of 0.21 ± 0.03 mm^−1^ and median of 0.20 mm^−1^ for HN and mean 0.25 ± 0.03 mm^−1^ and median of 0.26 mm^−1^ for pelvis/spine. The modified version of the algorithm showed a mean of 0.28 ± 0.02 mm^−1^ and median of 0.29 mm^−1^ for HN and 0.29 ± 0.01 mm^−1^ and median of 0.29 mm^−1^. We assume stationarity of SR in the image, and while this is not strictly true since resolution decreases with increasing distance from isocenter, the non‐stationary effect is negligible compared to our measurement uncertainty. Secondly, we assume that there is no difference in reconstructed image resolution due to material composition differences between phantom and patient. This assumption is true for linear reconstruction (filtered back‐projection), which was used in the study. This assumption may not necessarily hold for non‐linear reconstruction: iterative or deep‐learning reconstruction.

### 3.3 Filter sensitivity study

The number of ESF curves eliminated and the resulting accuracy of the method is presented in Table 3 and presented along an example invalid ESF curve removed by each of the filters.

**TABLE 3:** An in‐depth analysis of the effects of each filter on the f50 estimation.

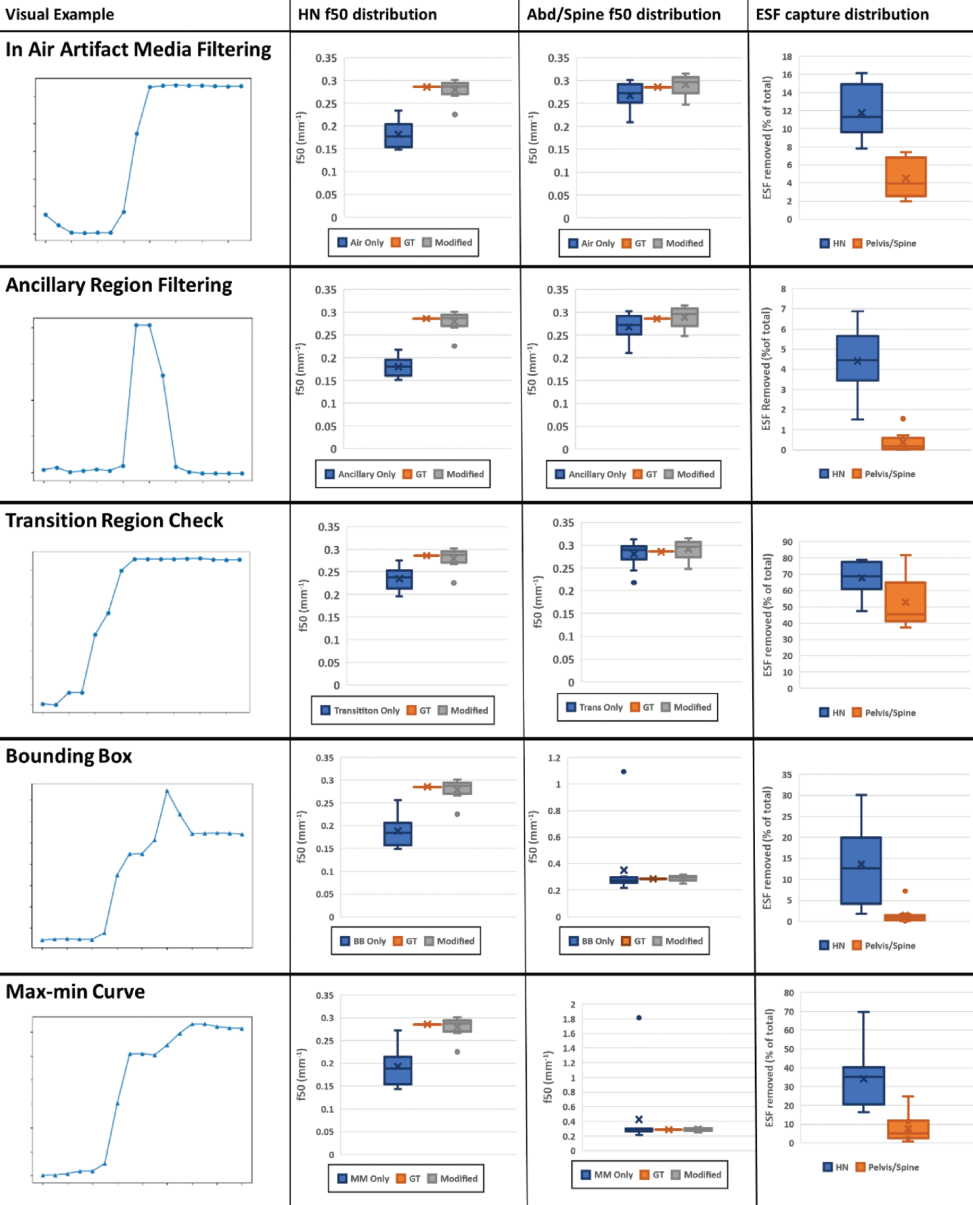

*Note*: Column 1 shows a representative example of the types of ESF curves the filter tends to remove, extracted from a spine patient in the dataset. Column 2 and 3 show the resulting f50 distribution for the HN and Abd/Spine patient data, respectively. Column 4 shows the percentage of total curves, this filter removes when used alone. We only look at the ESF curves that are in‐slice (± 10° normal to the XY plane). The clothing filter is also excluded from this study. The visual examples show the HU progression of each ESF sample from in‐air to hypothetically in‐tissue, and do not show the actual distance in tissue which may vary from point to point. Each filter is designed to handle a set of types of ESF curves such as the presence of artifacts in the air‐tail of the curve (in air artifact media filter). Additionally, we handle ancillary regions, such as armpits or nose using the ancillary region filter. Finally, the transition region filter eliminates ESF's where the transition region does not occur roughly in the center of the ESF. Post‐centering we look for oddities such as spikes in the ESF in tissue using the bounding box and max‐min filters.

The results of the study of mesh resampling are shown in Figure 5.

FIGURE 5The median percentage difference of the algorithm approximation from ground truth as a function of the resampling coefficient.

**Figure:**
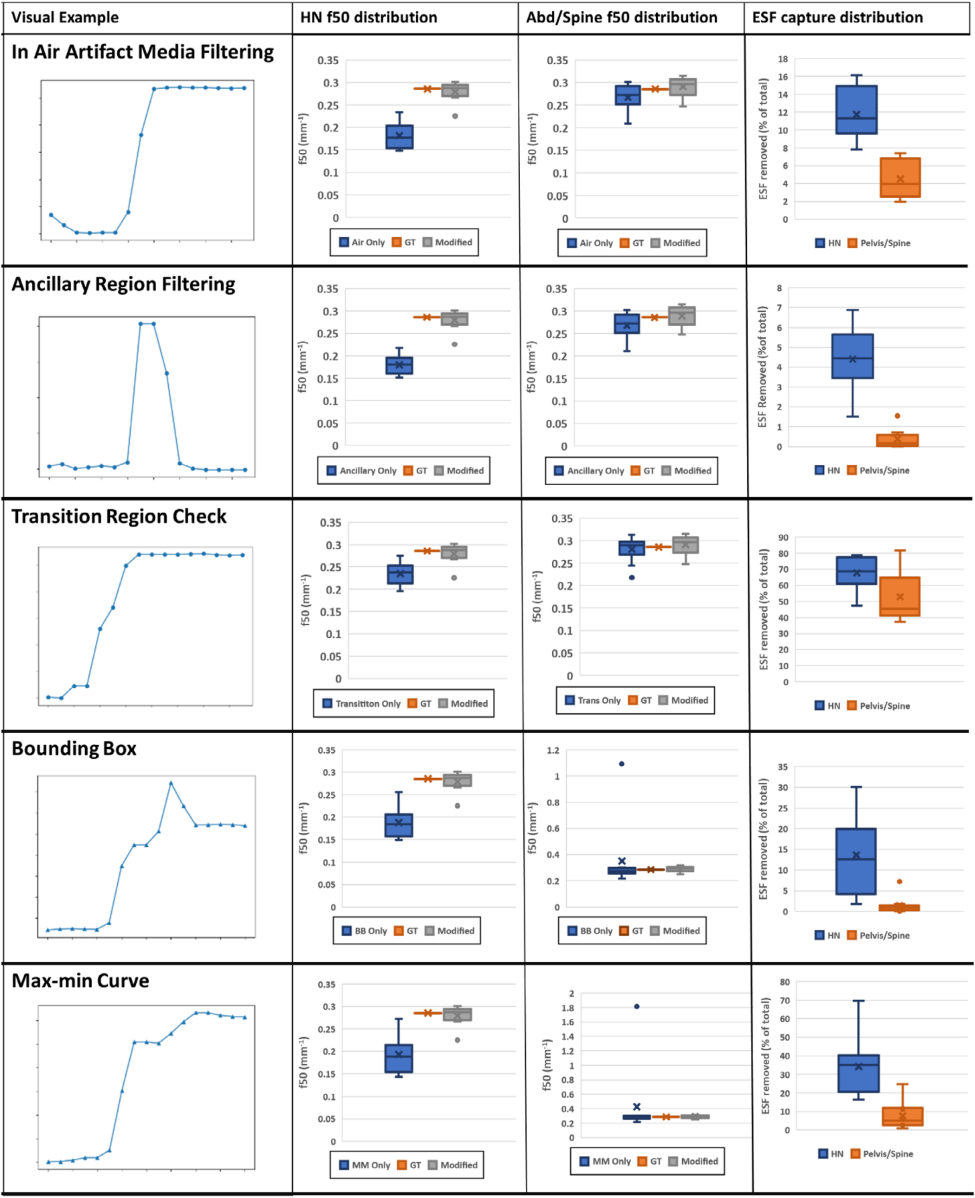

**Figure:**
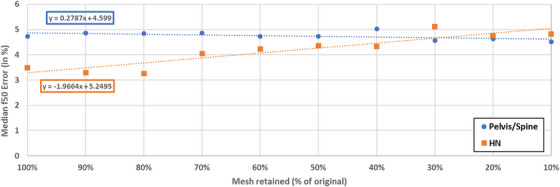

## 4 DISCUSSION

In summary, in this work we replicated the SR estimation algorithm introduced by Sanders et al. ^10^ and then improved the algorithm with new surface meshing algorithms and edge spread function filters in order to improve accuracy in radiotherapy CT simulation, including HN cases. We compared the performance of our modifications as compared the original work on cylindrical and anthropomorphic phantoms as well as a clinical dataset. Finally, we performed an evaluation of the individual filters as well as mesh quality on the algorithm's accuracy. Overall, we found that while our improvements improve the results certain imaging conditions still present a challenge to both versions of the algorithm.

The presented method is expected to be helpful in auditing SR in individual CT exams for which a corresponding phantom QC test is unavailable or is inconvenient to perform. An example situation is radiotherapy treatment planning using CT simulation imaging performed at a remote location.

The following discussion describes specific aspects of the performance of the method. The initial testing on various phantom reconstructions concurs with the results of the reference work, the algorithm is indeed sensitive to reconstruction kernel, and is exemplary at estimating SR effectively with commonly used kernels. When sharper kernels (f50 > 0.60 mm^−1^) are used, the resulting noise amplification led to over‐rejection of ESF curves in the modified algorithms, and the f50 estimate becomes unreliable. Trimming the in‐air and in‐tissue tails after the oESF was generated minimized this sensitivity in the reconstructions with the very sharp kernels in the phantom datasets, but despite this the resulting f50 is unreliable due to noise and over‐filtering due to our modifications. These results agree with the hypothesized limitations of this approach stated in the reference work and although this was observed, these reconstruction kernels have a higher sharpness according to f50 than typically used in radiotherapy simulation.

**FIGURE 6:**
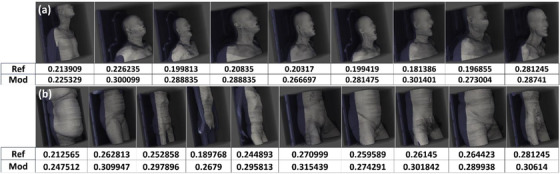
A visual representation of the patient testing sets for HN (a) and pelvis/spine scans (b). The volumetric data as well as the mesh generated using Otsu thresholding and marching cubes (in white) is visualized for both patient sets. Head and Neck meshes are shown post‐table crop, but prior to any additional cropping, and faces have been blurred due to privacy concerns. A table is provided underneath each set denoting the spatial resolution f50 value generated using the reference algorithm (top row) and the modified algorithm (bottom row).

The reference algorithm performed as expected on the clinical pelvis/spine datasets, with a slight tendency to underestimate the ground‐truth f‐50 value, as reported in the reference work. Furthermore, both algorithms had a smaller f‐50 as compared to the ground truth in the case of larger patients, where most of the samples fell outside of the 16‐cm radial bin (Figure 6). In the HN dataset, the modified version of the algorithm performed much better, due to the addition of the new filters, Taubin smoothing, and sampling under the mask ensuring only the best ESF curves are passed to the aggregation stage.

**FIGURE 7:**
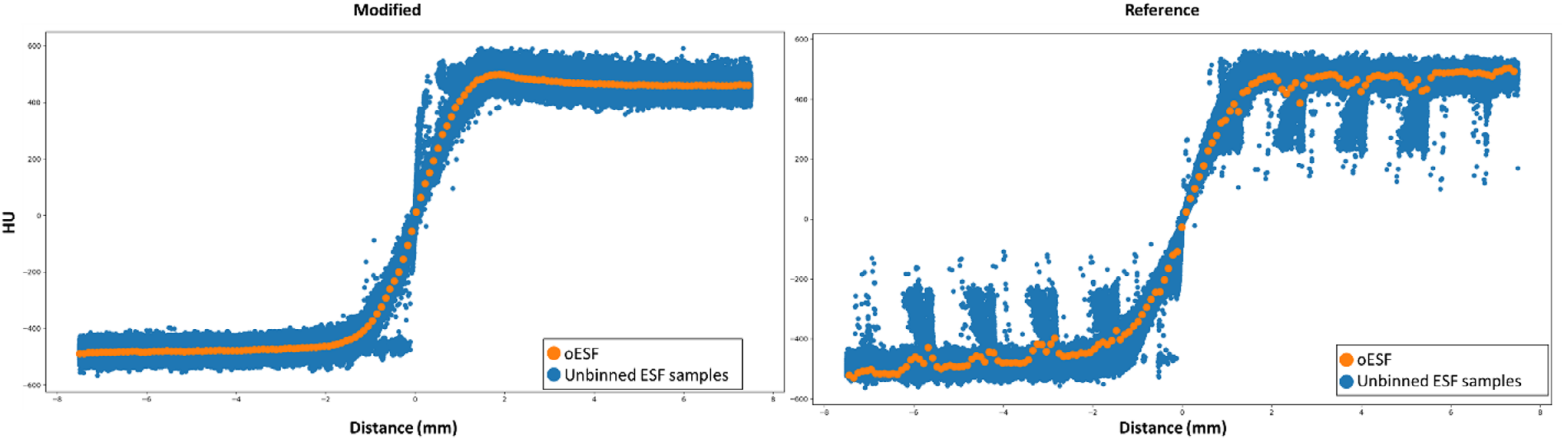
A comparison of the oESF from the modified and reference algorithms along with the first 1 million samples from the individual ESF points before binning, extracted from a head neck patient. Both oESF's have been conditioned. The reference algorithm tends to accept a large number of curves that pass through the mask, passing the clothing filter, and distorting the final oESF leading to inaccuracies in the SR readings in HN patients.

**FIGURE 8:**
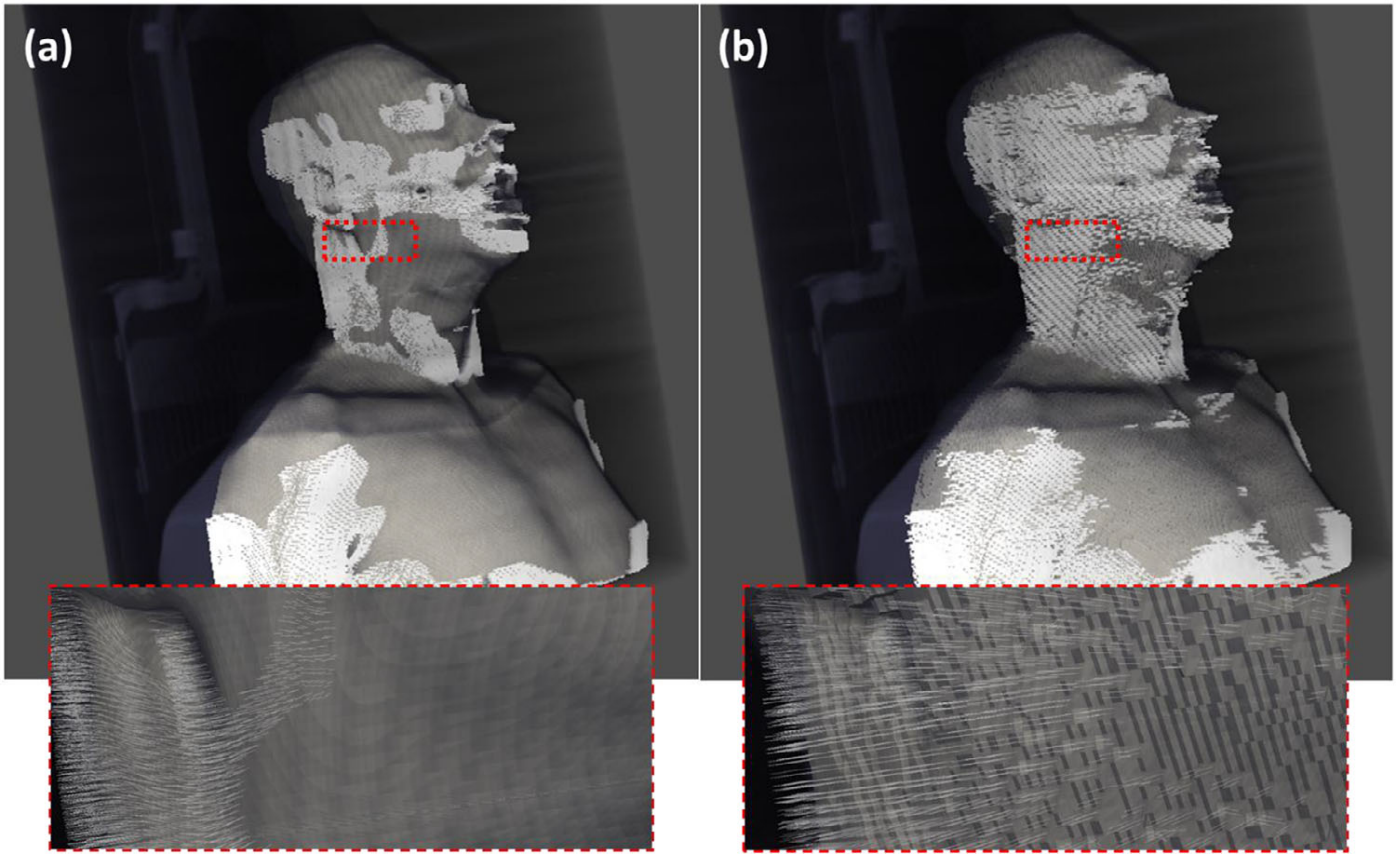
An example of a mesh with (a) and without (b) smoothing both with insets of approximately the same area around the ear. The smoothing operation reduces the size of the individual mesh elements by ≈15%, resulting in a proportional increase of the number of samples. This also results in a smaller inter‐sample distance, but minimizes the number of jagged cubic faces resulting in overall improvement in sample quality.

Additionally, as stated in the reference work, mesh generation can also be a challenge for the algorithm. This is particularly prevalent in HN cases, where facial features and transitions from shoulders tend to be very sharp resulting in virtually no samples in those areas, and what samples are left tend to pass through the immobilization device (Figure 7). This is not an issue in pelvis/spine CT simulation scans where patient positioning tends to lead to an abundance of flat areas of skin where the sampling strategy and mesh quality does not matter and all the samples pass the ±10° filter, consequently both versions of the algorithm perform well on areas of the mesh where curvature is low, such as chest, stomach and legs. Taubin smoothing and mesh truncation was essential in reducing the number of regions in the mesh where curvature increases sharply over just a few mesh elements while preserving the volume captured by the mesh. As shown in Figure 8, the smoothing operation does reduce the size of the mesh elements by approximately 15% resulting in a proportional increase in the number of total aggregated samples. Some improvement is due to the decrease in the number of jagged edges due to the voxelization from the thresholding method resulting in an overall improvement of the individual ESF quality. In the HN datasets, due to the presence of immobilization devices, a large number of the samples collected from the head and shoulder regions of a patient were discarded due to intersection with immobilization devices. The truncation procedure focuses the algorithm on the regions where intersections are less likely to occur, leading to further improvement in accuracy for HN patients.

The in‐air artifact media filter performed similarly to the clothing filter but tended to identify fluctuations where the raw HU value did not exceed –925 H.U., but fluctuated in amplitude, nevertheless. The ancillary region filter would identify ESF curves that would otherwise go through onto the centering stage of the algorithm. Depending on the length of the tail, such curves also passed the centering stage since the tail replacement portion of the algorithm only looked for the ascending portion of the curve to find the edge transition. There are many such ESF curves in the HN CT scans as shown in the nose, ear and mouth regions (Figure 8). The transition region filter removes curves that have unequally short or long tail sections, preventing problems further down the pipeline during the centering stage. These curves were usually artifacts of the meshing process caused either by the HU amplitude or mesh smoothing, resulting in displacement of the air–skin interface by a few voxels. Both post‐centering filters exist primarily to deal with outliers as a result of noise from sharper reconstruction kernels, regions where the skin is thin and the ESF transitions into bone resulting in a preserved transition region, but incorrect tail replacement. These filters allow for accurate estimation of the MTF across the range of CT simulation acquisition parameters, reconstruction parameters, and patient positions encountered in the clinic.

As the mesh is down‐sampled the accuracy of the algorithm on HN CT decreases, but for scans of the pelvis and spine the accuracy remains constant. Furthermore, there is some additional error due to new behavior in the mesh. One important filter in the original work was the in‐slice filter, which ensured that each ESF existed within a single slice by making sure that the angle of the Bresenham line was ±10°. Resampling the mesh resulted in samples passing the filter but transgressing the artificial slice borders, as the triangle centers now exist very close to slice borders, resulting in some jitter in accuracy. Overall, keeping 50% of the mesh was an acceptable option for running the algorithm when computational resources are limited, but retaining 100% of the mesh guarantees the lowest error.

There were some limitations to our study. One limitation of the method is the monotonicity constraint, which may result in MTF measurement error for reconstruction filters that have truly non‐monotonic MTF such as filters with edge‐enhancement. Additionally, while our algorithm has shown good results in approximating the gold standard SR for scans of the HN, we have not thoroughly tested our sampling strategy on immobilization devices of different types or from different vendors. Certain vendors produce immobilization devices that are specifically designed to adhere to the patient's head as closely as possible, and in those cases the modified algorithm may not be as accurate due to over filtering. A more consistent strategy of sampling between the sampling cells may be more appropriate. More novel methods might be needed for solid HN immobilization devices with no mesh structure. Additionally, we have not tested the algorithm in clinical scenarios with a variety of reconstruction filters and different voxel sizes, only in test phantoms. While the performance of the algorithm was stable for a cylindrical phantom, this performance may not translate to clinical data, therefore further study is needed.

## 5 CONCLUSION

In this study, the experimental method of CT image SR measurement was tested, validated, and improved for use in a range of clinical radiotherapy CT simulation procedures. Both algorithms were tested against the ground truth in cylindrical and anthropomorphic phantoms as well as a variety of clinical acquisitions. The reference algorithm was improved through the implementation of additional filters that function similarly to the Swiss cheese model to filter out invalid ESF samples. Although each individual filter does not remove every invalid ESF sample, the combination of filters is effective in producing a valid set of ESF samples, and therefore an accurate MTF measurement. The method may be robust to a variety of inter‐patient differences such as positioning and presence of immobilization devices. The presented method may be used as a surrogate of a traditional resolution measurement taken in phantom.

## AUTHOR CONTRIBUTIONS

The work was conceptualized by Laurence Court, Rick Layman and Ahmad Moiz. Pavel Govyadinov contributed to phantom and patient data analysis, algorithm development and testing. Rick Layman, Tucker Netherton, Ahmad Moiz and Aaron Kyle Jones performed the phantom data acquisition. Raymond Mumme performed the patient data curation. All authors contributed intellectual content the writing of the manuscript and had final approval.

## CONFLICT OF INTEREST STATEMENT

The authors have no conflict to disclose.

## Supporting information



Supporting Information

Supporting Information
