# Supplementary material for: Robust automated method of spatial resolution measurement in radiotherapy CT simulation images
Source: J Appl Clin Med Phys. 2025 Feb 13;26(3):e70006. doi: 10.1002/acm2.70006 (PMC11905253; doi:10.1002/acm2.70006)

PURPOSE / OBJECTIVES

Image quality can vary in CT images used for radiotherapy planning. To ensure accurate treatment plans, it is necessary to objectively quantify image quality parameters like spatial resolution on a patient-specific basis. Advances in automatic radiation treatment planning (e.g. target and OAR segmentation) have heightened the need for automated quality control on treatment planning imaging. A previously published reference algorithm [1] has demonstrated automatic spatial resolution measurement in clinical CT images, but its reliability over varying reconstruction kernels and patient immobilization scenarios has not been demonstrated. We have modified the reference algorithm for robustness to these variations. The resulting spatial resolution quality test may be used in an automated workflow to minimize the risk of automated planning errors due to technical image quality variation.

MATERIAL & METHODS

- A reference algorithm for automated assessment of spatial resolution was implemented in Python and modified with a different surface mesh structure and robust filtering of edge response samples. Ground truth was measured using the Friedman algorithm [2]
- The modulation transfer function (MTF) f50 metric was measured using both the modified and reference algorithms on several datasets:
  - The ACR CT QC phantom was imaged using a Siemens SOMATOM Definition Edge Scanner and reconstructed using 61 different kernels.
  - Anthropomorphic phantoms with commonly present extrinsic objects, such **as immobilization devices, blankets and fiducial markers** were imaged in a setup mimicking thorax, abdomen & pelvis and head & neck CT simulations. Phantoms scans were collected using the Phillips Brilliance Big Bore and accompanied by CDTI phantoms for ground truth resolution measurement.

RESULTS

Both the reference and modified algorithms were accurate when ground truth MTF f50 was within the range of 0.2–0.7 cm^{-1}. 10 reconstruction kernels were deemed as outliers. Removing the outliers, the accuracy of both algorithms was within 9.67% with RMS of 3.81% and 10.23% for modified and reference respectively. The modified algorithm showed no discernible bias over the range of reconstruction kernels.

The reference algorithm had errors of 43.1%, 4.9% and 59.4% from the ground truth, largely attributed to the axillary region and the immobilization devices. In comparison, the anthropomorphic phantoms the modified algorithm was more robust to errors and had improved accuracy, with errors of 4.12%, 3.30% and 3.31% for abdomen & pelvis, thorax, head & neck respectively.

In the process of generalizing the algorithm to handle a variety of patient imaging setup scenarios, a new set of filters needed to be developed. The head & neck site required effective mitigation of the immobilization device and a special strategy of sampling in areas under the immobilization device.

- A reference algorithm for automated assessment of spatial resolution [1] was modified and generalized to function on multiple clinical protocols.
- New modifications adaptive mesh generation included the removal of ESF samples with breaks in the derivative (i.e. more than one medium transition) and ESF samples with a poor logistic function fit.
- New filtering and sampling strategies in the modified algorithm yielded an improved accuracy in anthropomorphic phantom datasets of 4.12%, 3.30% and 3.31% for abdomen & pelvis, thorax, head & neck respectively.

ALGORITHM SUMMARY

Figure 1: Figure demonstrating the reference algorithm for the MTF f50 measurement. The patient body is segmented and the skin surface is sampled using the previously published method (a). The section of each individual sample inside the tissue are replaced with the section in air to generate symmetry (b). The curves are centered such that the inflection point of every curve is at (x,y) = (0,0) (c). And Oversampled ESF curve is generated by binning all the points with respect to 10% of pixel spacing (d), and finally the MTF is generated from the oversampled ESF curve (e)

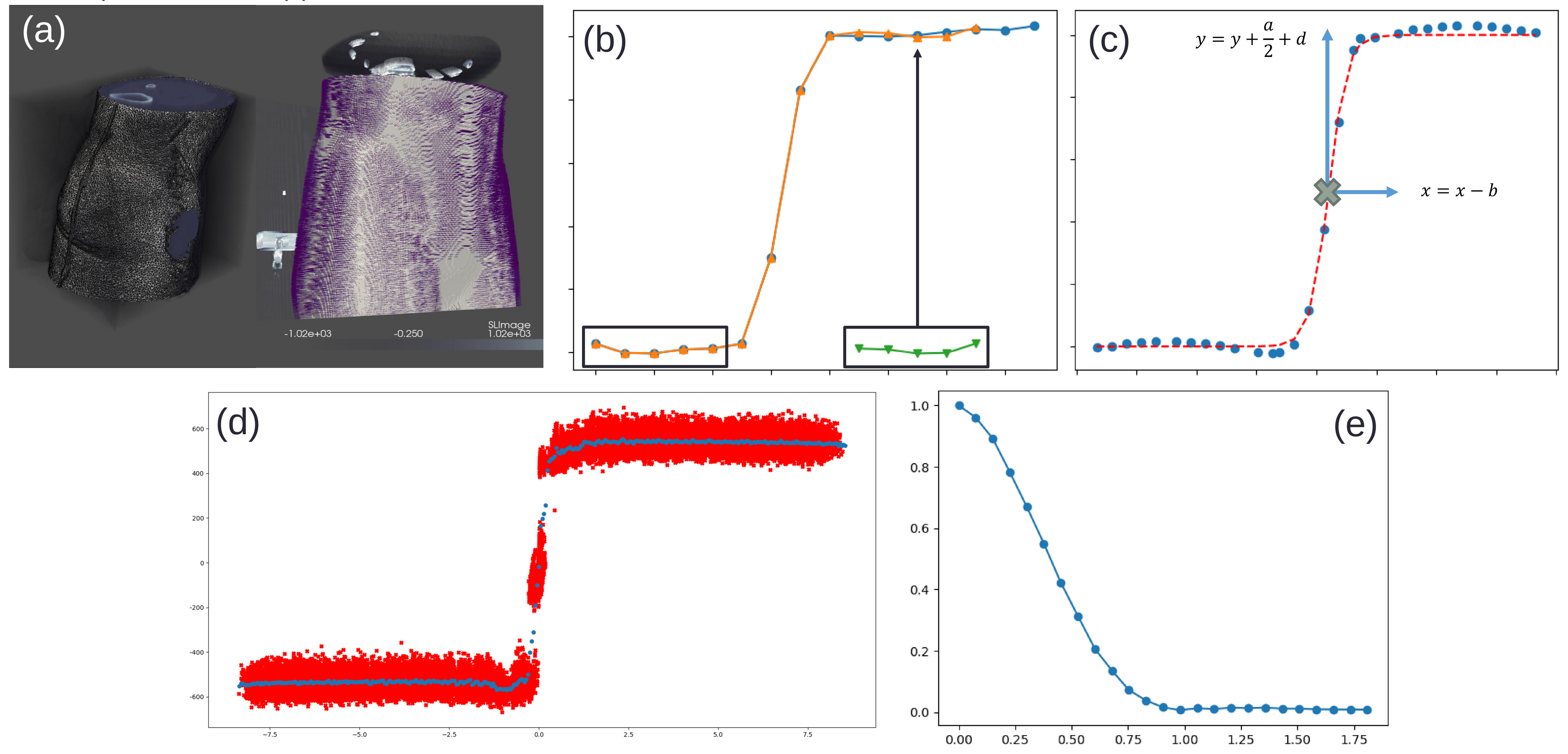

RESULTS

Figure 2: The anthropomorphic phantom during imaging (left) and accompanying segmentation of the skin-air interface and immobilization device (right). Head and neck scans required a change in the sampling strategy to sample under the mask

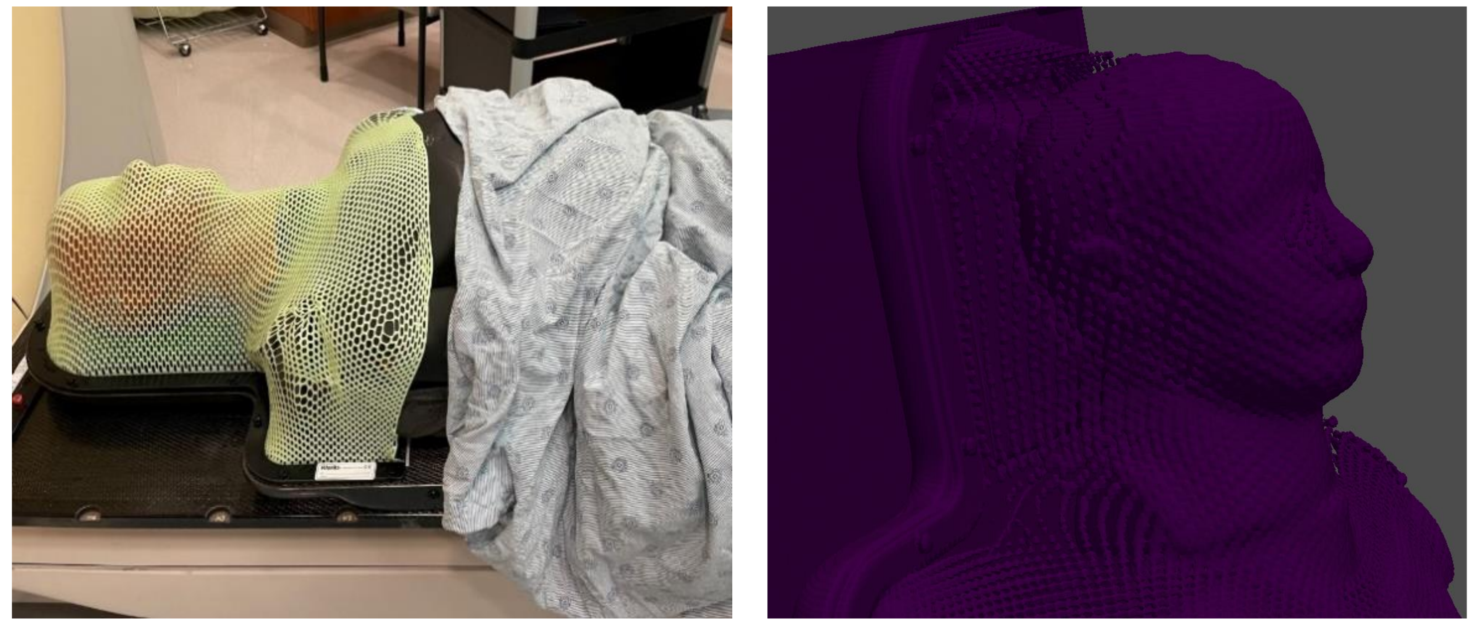

Figure 3: Accuracy of the modified and reference resolution measurement algorithms over a range of image reconstruction kernels with varying spatial resolution (modulation transfer function f50 value). Mean error (solid) and ± 2STD (dashed) lines are also plotted. The typical f50 for simulation performed with field of view of 50cm is labeled on the plot in green.

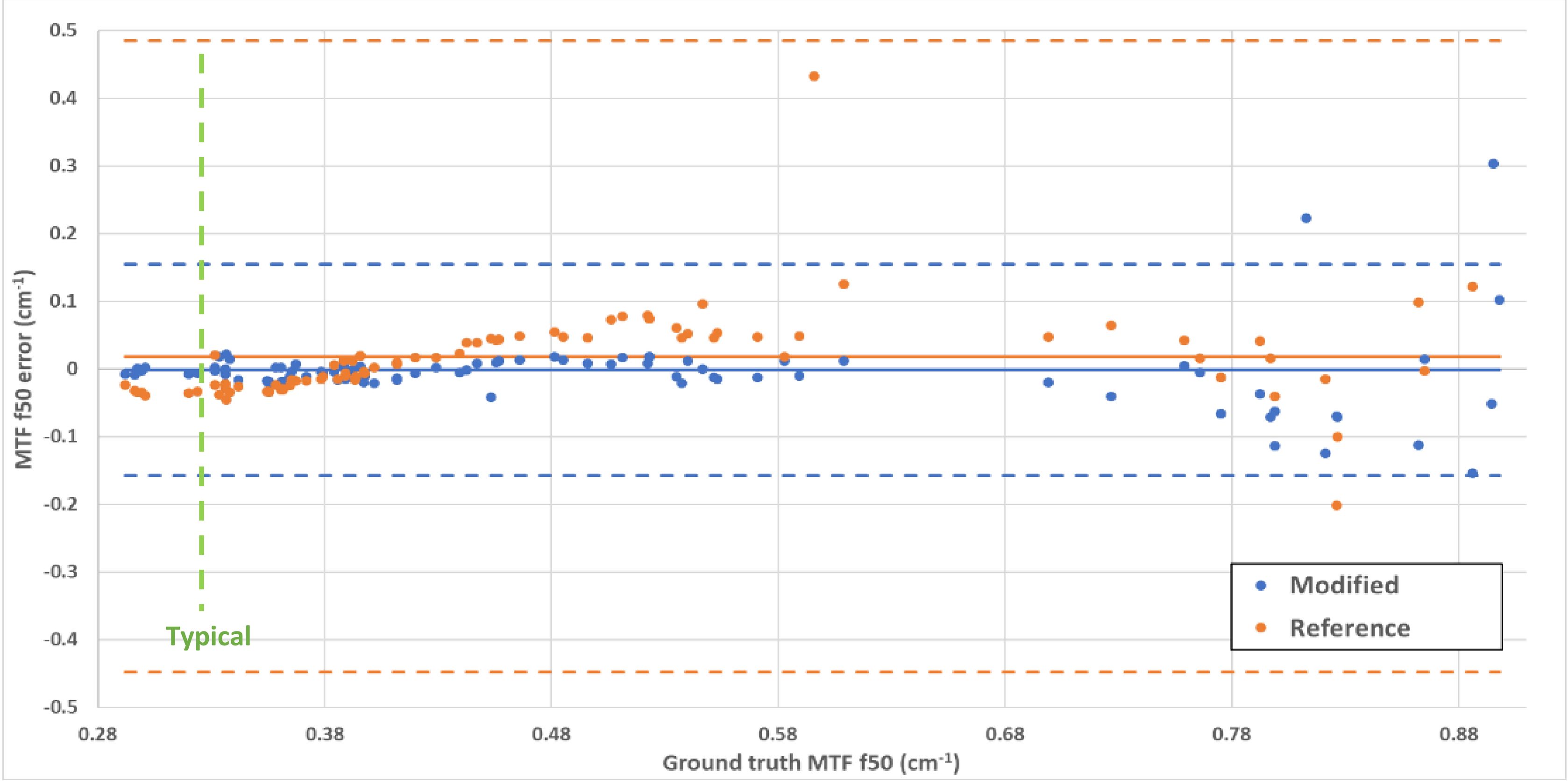

Supplement: Supplementary file 1 — Supporting Information [file ACM2-26-e70006-s001.pdf]
