# Supplementary material for: Robust automated method of spatial resolution measurement in radiotherapy CT simulation images
Source: J Appl Clin Med Phys. 2025 Feb 13;26(3):e70006. doi: 10.1002/acm2.70006 (PMC11905253; doi:10.1002/acm2.70006)
Supplement: Supplementary file 2 — Supporting Information [file ACM2-26-e70006-s002.docx]

**1 | APPENDIX A: LIST OF ANALYZED KERNELS**

Table 1:Table of all kernels used to reconstruct the cylindrical phantoms scanned using the Abdomen and AbdSeq phantoms.

| Bf37 | Bf37 | Bf37 | Bf37 | Bf37 | Bf37 |
| --- | --- | --- | --- | --- | --- |
| Bf39 | Bf39 | Bf39 | Bf39 | Bf39 | Bf39 |
| Bf42 | Bf42 | Bf42 | Bf42 | Bf42 | Bf42 |
| BI57 | BI57 | BI57 | BI57 | BI57 | BI57 |
| Br32 | Br32 | Br32 | Br32 | Br32 | Br32 |
| Br34 | Br34 | Br34 | Br34 | Br34 | Br34 |
| Br36 | Br36 | Br36 | Br36 | Br36 | Br36 |
| Br38 | Br38 | Br38 | Br38 | Br38 | Br38 |
| Br40 | Br40 | Br40 | Br40 | Br40 | Br40 |
| Br43 | Br43 | Br43 | Br43 | Br43 | Br43 |
